# Two-Year Experience of a Center of Excellence for the Comprehensive Management of Non-Small Cell Lung Cancer at a Fourth-Level Hospital in Bogota, Colombia: Observational Case Series Study and Retrospective Analysis

**DOI:** 10.3390/jcm13226820

**Published:** 2024-11-13

**Authors:** Luis Gerardo García-Herreros, Enid Ximena Rico-Rivera, Olga Milena García Morales

**Affiliations:** Fundación Santa Fe de Bogotá Centro de Cuidado Clínico de Cáncer de Pulmón, Bogotá 110111, Colombia

**Keywords:** lung neoplasms, carcinoma, non-small-cell lung, immunotherapy, radiotherapy, survival, drug-related side effects and adverse reactions

## Abstract

**Background:** This study aimed to provide a comprehensive analysis of 56 patients admitted to the Lung Cancer Clinical Care Center (C3) at Fundación Santa Fe de Bogotá (FSFB) between 2 May 2022 and 22 April 2024. The focus was on demographic characteristics, smoking history, comorbidities, lung cancer types, TNM classification, treatment modalities, and outcomes. **Methods:** This observational case series study reviewed medical records and included patients over 18 years with a confirmed diagnosis of non-small cell lung cancer (NSCLC). Data were collected and analyzed for demographics, comorbidities, treatment types, biomolecular profiling, and survival rates. Ethical approval was obtained, and data were anonymized. **Results:** The mean age was 71.8 years with a female predominance (53.6%). A history of smoking was present in 71.4% of patients. Adenocarcinoma was the most common type (75.0%), followed by squamous cell carcinoma (19.6%). At admission, the most frequent TNM stages were IA2 (17.9%) and IVA (16.1%). One-year survival was 68.8%, and 94.3% of stage I–IIIA patients underwent PET scans. Biomolecular profiling revealed 69.2% non-mutated EGFR, 90.4% ALK-negative, and various PDL-1 expression levels. Immunotherapy was received by 91.4% of patients, with Alectinib and Osimertinib being common. Grade III–IV pneumonitis occurred in 5.4% of patients. **Conclusions:** The study’s findings align with existing literature, highlighting significant smoking history, common adenocarcinoma, and substantial use of immunotherapy. Limitations include the observational design, small sample size, and short follow-up period, impacting the generalizability and long-term outcome assessment. Future research should address these limitations and explore longitudinal outcomes and emerging therapies.

## 1. Introduction

Lung cancer, or bronchogenic carcinoma, arises in the lung parenchyma or bronchi and stands as one of the principal causes of cancer-related mortality both globally and in the United States. Since 1987, it has surpassed breast cancer as the leading cause of cancer death in women, with an estimated 225,000 new cases and 160,000 deaths occurring annually in the U.S. alone [1,2]. In 2021, projections from the International Association for the Study of Lung Cancer (IASLC) indicated 235,760 new cases and 131,880 deaths within the U.S. [3]. In Colombia, approximately 4000 new cases are diagnosed each year, with a high proportion (72%) presenting at advanced or metastatic stages, contributing to a low 5-year survival rate of 8.7% [4]. Lung cancer incidence has increased sharply over the 20th century, largely attributable to the rise in tobacco use [1,2]. Globally, lung cancer is the leading cause of cancer death and the second most common cancer after breast cancer, comprising around 12.4% of all cancer diagnoses and responsible for approximately 1.8 million deaths annually [5,6,7,8,9]. In developing regions, incidence rates have grown significantly, with nearly half (49.9%) of new cases now occurring in these areas [10]. In the U.S., lung cancer mortality is generally higher in men, with African American men experiencing higher age-adjusted mortality rates compared to Caucasian men, though no significant racial differences are noted among women [11]. Tobacco smoking remains the primary cause of lung cancer, linked to roughly 90% of cases [11]. Additional risk factors include asbestos exposure, radon, passive smoking (which increases risk by 20–30%), previous radiation therapy (particularly for cancers like non-Hodgkin lymphoma and breast cancer), and specific occupational exposures to metals and hydrocarbons [11,12,13,14]. Radon, especially in mining settings, and residential radon exposure have both been associated with increased lung cancer mortality, particularly among smokers [6,7].

In Colombia, fragmented healthcare access for lung cancer patients often results in delayed diagnosis, suboptimal treatment, and insufficient follow-up, leading to worse patient outcomes and heightened mortality. These barriers, coupled with frequent hospitalizations and prolonged treatments, contribute to a significant economic burden on the healthcare system [4]. In response to these challenges, Fundación Santa Fe de Bogotá (FSFB) established the Lung Cancer Clinical Care Center (C3) in 2020. This center aims to improve lung cancer outcomes through a model of high-quality care based on four pillars: advanced clinical management, high-impact metrics, patient-centered care, and research. This case series describes C3’s lung cancer management experience and presents the institutional registry of non-small-cell lung cancer (NSCLC) patients. The registry is designed to systematize case records, elevate care standards, and provide a foundation for ongoing research in lung cancer [4].

## 2. Materials and Methods

This is a descriptive observational study of a case series. The study population consisted of patients enrolled in the Lung Cancer Clinical Care Center (C3) at Fundación Santa Fe de Bogotá (FSFB) from the start of systematic data collection on 2 May 2022, until the last patient enrollment on 22 April 2024.

### 2.1. Selection Criteria

#### 2.1.1. Inclusion Criteria

The inclusion criteria for this study are the same as those of the C3 Lung Cancer Center at FSFB and included:

- Patients over 18 years old.
- Patients with a confirmed diagnosis of primary lung cancer, including non-small cell lung cancer (NSCLC), of all histological subtypes and stages according to the most recent version of the TNM classification.
- Patients initially diagnosed at FSFB or referred from other institutions with confirmed pathology for continued therapy at C3.
- Patients for whom protocol continuity can be ensured at the institution.

#### 2.1.2. Exclusion Criteria

The exclusion criteria for this study are the same as those of the C3 Lung Cancer Center at FSFB and included:

- Patients with lung metastases from a primary tumor located elsewhere.
- Patients with lung cancer and a life expectancy of less than 6 months (e.g., those with high tumor burden, refractory to multiple treatment lines, or in irreversible functional decline treated outside the C3 protocol).
- Patients who refused to receive comprehensive care at FSFB.

### 2.2. Patient Evaluation and Management Definition

After confirming the histopathological diagnosis of non-small-cell lung cancer (NSCLC) with the appropriate biomarkers, either through biopsy or surgical resection (pTNM), the C3 proceeded with disease staging. This was carried out using the 8th edition of the TNM classification to define the stage and guide the appropriate treatment. This record was used for the purpose of this research. Staging was performed non-invasively with a chest CT scan and, in all cases, a PET scan, along with an MRI of the brain in special cases to assess the probability of metastasis. Invasive staging was performed using procedures such as mediastinoscopy or endobronchial ultrasound (EBUS) if the chest CT or PET scan revealed mediastinal involvement or probable involvement of N1 lymph nodes.

Tumor size alone was not a criterion for invasive staging, as this had a level C evidence grade, and each case was individually evaluated [15]. According to the experience of the C3, central tumors dependent on lobar bronchi always required invasive staging. Initial pathological samples used for histopathological diagnosis underwent at least biomarker testing for ALK, PD-L1, and EGFR. Depending on the specific characteristics of each patient, additional biomarkers were tested. In surgical specimens, criteria for pathological staging were evaluated, and if necessary, the pathology team reclassified the tumor. New biomarker tests could be performed based on available treatments and the multidisciplinary team’s decisions. Upon confirming the NSCLC diagnosis histologically, the treating physician determined the most beneficial treatment for the patient, which could include oncological management, surgical management, or a combination of both.

#### 2.2.1. Tumor Classification by Histological Subtype

The histopathological classification of lung cancers was based on cellular and molecular subtypes, which is essential for diagnosis and management. The World Health Organization (WHO) 2021 lung tumor classification system divides lung cancers as follows [16]:

- Adenocarcinomas;
- Squamous cell carcinomas;
- Precursor glandular lesions;
- Adenosquamous carcinomas;
- Precursor squamous lesions;
- Large-cell carcinomas;
- Sarcomatoid carcinomas;
- Pulmonary neuroendocrine neoplasms;
- Salivary gland-type tumors;
- Neuroendocrine tumors;
- Neuroendocrine carcinomas;
- Other epithelial tumors.

According to the WHO, identifying histological characteristics, measuring the depth of invasion, and the mode of spread have prognostic value [16]. For example, tumor spread through airways is associated with a higher recurrence rate after limited resections and should be reported in pathology evaluations [16]. The WHO has removed previously described subtypes such as clear cell, rhabdoid, and signet ring from its latest classification, as these appear to be cytological features that can occur in any adenocarcinoma [16]. The WHO classification emphasizes immunohistochemical staining to classify cancers that may not have typical cytological features under optical microscopy [16]. In the WHO 2015 classification system, poorly differentiated carcinomas were reclassified as squamous cell carcinomas if they expressed p40; as solid adenocarcinomas if they expressed thyroid transcription factor 1; and as neuroendocrine carcinomas if positive for chromogranin and synaptophysin [17].

Given that C3 only includes patients with NSCLC within the inclusion criteria, only histological subtypes corresponding to this type of cancer were considered for this study.

#### 2.2.2. Lung Cancer Staging (TNM System) in the Studied Population

Staging was conducted using the TNM classification system in its last version [15] to plan treatment and guide prognosis for each patient. This classification was used to evaluate the anatomical extent of the disease both clinically and from a histopathological point of view.

#### 2.2.3. Evaluation of Biomarkers in the Studied Population

The pathophysiology of lung cancer is highly complex and not fully understood. It is believed that repeated exposure to carcinogens such as cigarette smoke leads to pulmonary epithelial dysplasia. Continued exposure can induce genetic mutations, thereby affecting protein synthesis [18]. This disruption causes cell cycle dysregulation and fosters carcinogenesis. The most common genetic mutations involved in lung cancer include MYC, BCL2, and p53 for small-cell lung cancer (SCLC), and EGFR, KRAS, and p16 for non-small-cell lung cancer (NSCLC) [19,20].

These biomarkers are crucial because the primary treatment for metastatic NSCLC relies on identifying specific driver genetic mutations. These include alterations in the epidermal growth factor receptor (EGFR) and rearrangements in the anaplastic lymphoma kinase (ALK) gene [19,20,21]. Additionally, PD-L1 expressions have been associated with increased tumor proliferation, cancer aggressiveness, and reduced survival in NSCLC patients, particularly those diagnosed with adenocarcinoma [22].

Therefore, during the histopathological diagnosis of lung cancer at C3, it was crucial to conduct a minimum biomarker analysis including ALK, PD-L1, and EGFR using the initial pathological sample, especially for patients in stages III and IV of the disease, to ensure targeted therapy [21,22]. The assessment of these biomarkers was considered for research purposes. This initial evaluation could be expanded to include other biomarkers based on each patient’s specific characteristics. Thus, whenever the treating physician requested evaluation of biomarkers other than those mentioned, these were documented in the study’s results section.

#### 2.2.4. Functional Impact of Oncologic Disease (ECOG Performance Status) and Its Evaluation in the Study Population

Clinical research involving cancer patients necessitates the use of standardized criteria to measure the disease’s impact on patients’ ability to perform daily activities, known as the patient’s functional status. The Eastern Cooperative Oncology Group (ECOG) Performance Status Scale corresponds to a well-established system widely referenced in the literature for such measurements [23].

This scale describes a patient’s level of functioning in terms of their ability to self-care, perform daily activities, and engage in physical activities (e.g., walking, working, etc.) [23]. Researchers worldwide consider the ECOG Performance Status Scale when planning oncologic clinical trials to study new treatments. This numerical rating system helps define the patient population for the trial and guides physicians enrolling patients in these studies. It also allows physicians to monitor changes in a patient’s functional level resulting from treatment during a clinical trial [23]. The updated ECOG Scale was used in this study to evaluate the impact of the disease on the quality of life of the participants.

For study purposes, the ECOG Performance Status scores were recorded and analyzed at the initial assessments of the patients to ascertain the presence and potential magnitude of differences in patient functionality between the time of admission to C3 and their current status.

### 2.3. Study Procedures

#### 2.3.1. Data Collection, Tabulation, and Data Cleaning

As part of the routine clinical practice at C3, all necessary information to meet the study objectives was collected from each patient’s medical records. At FSFB, a university hospital, all patients must sign an informed consent allowing the use of their data for research, in accordance with approval from the Institutional Research Ethics Committee.

Subsequently, an epidemiologist and hospital physician from FSFB with research experience reviewed all C3 medical records starting from 2 May 2022. She tabulated all the information into a Microsoft Excel^®^ database specifically designed for this study, which was further analyzed for the final report. This database incorporates all variables outlined in the study protocol.

#### 2.3.2. Description of Potential Biases and Measures to Control Them

As an observational case series study, the primary bias that could arise is information bias, which occurs when collected information about cases is inaccurate or incomplete. This may result from errors in data collection, interpretation, or inadequate record-keeping. To mitigate and minimize the likelihood of this bias, the following strategies were implemented:

- Double review of medical records: Information from medical records was reviewed in duplicate to minimize errors in interpretation and recording.
- Training and expertise of personnel: The data collection process was overseen by a hospital physician from C3, an epidemiologist experienced in research, data collection, and conducting observational studies.

#### 2.3.3. Statistical Analysis

For this study, data description included qualitative and quantitative variables, with corresponding frequency and percentage analysis for each category, as well as frequency distribution for variables of interest in qualitative data. Summary measures such as mean, range, and standard deviation were used for quantitative variables.

All statistical analyses were conducted using the latest versions of Microsoft Excel^®^ and SPSS.

#### 2.3.4. Ethical Aspects of This Study

This study was classified as minimal-risk research, according to definitions outlined in Resolution 8430 of 1993 by the Colombian Ministry of Health [24], which regulates clinical research in Colombia. Approval from the FSFB Research Ethics Committee was obtained to conduct this study (CCEI-16751-2024).

As an observational descriptive study, this research did not involve administering or modifying treatment regimens for participating patients. This study adhered to and respected established norms for research involving humans, as defined by national and international regulations, including the Declaration of Helsinki and its subsequent revisions [25].

Furthermore, all patients at FSFB, being a university hospital, provided informed consent upon admission to C3, voluntarily agreeing to the use of their data for research purposes. All information was handled confidentially. Results were presented in aggregate form to ensure anonymity and protect the identity of each participant.

## 3. Results

### 3.1. Baseline Characteristics and Smoking History

Fifty-six patients were admitted to C3 between 2 May 2022 and 22 April 2024. The age range was between 49 and 90 years, with a mean age of 71.8 years. Regarding gender distribution, 30 (53.6%) patients were female, while the remaining 26 (46.4%) patients were male.

In the analysis of the 56 patients, it was found that 71.4% (n = 40) were smokers at some point in their lives, while 28.6% (n = 16) never smoked. Additionally, none of the patients currently smoke, indicating a significant prevalence of smoking history, though without the presence of active smokers in the studied population (Table 1).

In the initial analysis of the patients’ functional condition, evaluated using the ECOG Scale, it was found that most patients had an ECOG level of 0 and 1, with 18 and 23 cases, respectively. Only one patient was classified as ECOG 2 at the time of admission. This result suggests that most patients maintained an adequate level of activity and could carry out light work.

### 3.2. Baseline Pathologies and Medications

In the analysis of the 56 patients, the most frequent pathologies were hypertension (21.4%), diabetes mellitus (14.3%), chronic obstructive pulmonary disease (COPD) (10.7%), ischemic heart disease (8.9%), dyslipidemia (7.1%), hypothyroidism (5.4%), previous cancer (3.6%), and chronic renal failure (3.6%), with various other pathologies representing 25.0%. The most frequently used medications were atorvastatin (21.4%), omeprazole (14.3%), metformin (10.7%), enalapril (8.9%), levothyroxine (7.1%), metoprolol (5.4%), insulin (3.6%), and aspirin (3.6%), with various other medications representing 25.0%. These results highlight the prevalence of chronic diseases such as hypertension and diabetes and the common use of medications for their control (Table 2).

### 3.3. Lung Cancer Type and TNM Stage

Regarding the type of lung cancer, 42 (75.0%) patients were diagnosed with adenocarcinoma, 11 (19.6%) patients with squamous cell carcinoma, 2 (3.6%) patients with poorly differentiated carcinoma, and 1 (1.8%) patient with infiltrating carcinoma with neuroendocrine differentiation (Table 3).

Regarding the TNM classification at the time of admission to C3, one patient was classified as stage IA1 (1.8%), ten patients as IA2 (17.9%), three patients as IA3 (5.4%), eight patients as IB (14.3%), one patient as IIA (1.8%), six patients as IIB (10.7%), six patients as IIIA (10.7%), four patients as IIIB (7.1%), two patients as IIIC (3.6%), nine patients as IVA (16.1%), and six patients as IVB (10.7%) (Table 3).

On the other hand, the current TNM stage at the time of the last visit to C3 was as follows: one patient was classified as stage IA1 (1.8%), nine patients as IA2 (16.1%), three patients as IA3 (5.4%), eight patients as IB (14.3%), one patient as IIA (1.8%), four patients as IIB (7.1%), six patients as IIIA (10.7%), four patients as IIIB (7.1%), two patients as IIIC (3.6%), nine patients as IVA (16.1%), and nine patients as IVB (16.1%). Disease progression was documented in only three of the 56 patients (Table 3).

### 3.4. One-Year Survival and PET Scan Results

The one-year survival analysis from diagnosis was conducted in 16 patients who completed one year of follow-up. The results indicate that 11 of these patients, representing 68.8% of the analyzed group, survived at least one year after the initial diagnosis. In contrast, five patients, equivalent to 31.2% of the group, died within the first year.

In the evaluation of PET scan use in patients diagnosed with stage I–IIIA cancer (35 patients), it was observed that a high percentage of these patients, specifically 94.3%, received a PET scan as part of their initial diagnostic evaluation. Two patients, representing 5.7%, did not undergo this diagnostic procedure.

In the combined analysis of patients diagnosed with stage I–IIIA cancer who received a PET scan, it was observed that of those who underwent the procedure, 24 patients, equivalent to 72.7%, obtained a negative result, indicating the possible absence of significant tumor activity or the effectiveness of previous treatments. On the other hand, nine patients, representing 27.3% of the evaluated group, showed positive results in the PET scan, suggesting the presence of active tumor activity or recurrence.

### 3.5. Surgical Procedures

Out of a total of 56 patients in the database, 39 patients, corresponding to 69.6%, underwent surgical procedures for lung cancer treatment. Of these 39 patients, 8 of them, representing 20.5%, had to undergo more than one surgical intervention at different times.

The analysis of surgical treatments in the database indicates that various surgical interventions were performed, each specifically tailored to the individual patient’s needs, including segmental lobectomies, wedge resections, mediastinoscopies with biopsies, and combinations of thoracoscopies with pleurodesis, among others. Each type of surgery was documented in a single instance, highlighting the personalization of surgical treatment. Most of these interventions (84.6%) resulted in an R0 resection, indicating no cancer cells were found in the resected tissue margins. Regarding postoperative recovery, 76.9% of patients did not require hospital readmission within 30 days post-operation, while 23.1% of patients were readmitted for various reasons such as infection, medication adjustments, and gastrointestinal problems, among others. This analysis shows effective surgical management, with a minority of cases experiencing significant postoperative complications.

### 3.6. Biomolecular Profiles and Biomarker Analysis

Fifty-two of the fifty-six patients included in this study underwent biomolecular analysis for the biomarkers EGFR, ALK, and PDL-1. This analysis was primarily conducted in patients with stage III to IV cancer, indicating that most of the evaluated sample received detailed biomolecular assessments to guide more personalized and targeted treatment decisions.

In the analysis conducted with 52 patients who underwent EGFR biomarker analysis, it was found that the majority, 69.2%, had non-mutated EGFR. Specific mutations were also identified, with 15.4% of patients showing the EX19DEL mutation, while the EX20INS and L858R mutations were present in 7.7% each.

In the ALK biomarker analysis conducted on 52 patients, it was observed that the majority, 90.4%, were ALK negative, indicating the absence of the ALK mutation in most of the evaluated patients. Additionally, 7.7% of patients were ALK positive, while 1.9% showed a weak positive ALK result.

Regarding the PDL-1 analysis conducted in 52 patients, it was found that 11.5% of patients showed 0% expression, while 34.6% exhibited less than 1% expression. Expressions of 1% and 3% were observed in 3.8% and 5.8% of patients, respectively. The expression of 5% was reported by 11.5% of patients. Other expressions, such as 10%, 20%, and 30%, were present in 5.8%, 7.7%, and 5.8%, respectively. Expressions of 35%, 40%, 70%, 80%, and 90% were less common, recorded in 1.9% of patients each.

### 3.7. Oncological Management, Therapies, and Adverse Events

Thirty-seven patients, equivalent to 66.1% of the evaluated sample, received oncological management. Of these patients, 91.4% received immunotherapy, with the most frequently used treatments being Alectinib in 11.11% of cases, followed by Osimertinib with 8.3%. Other medications had a more dispersed distribution, each representing 2.8% of the total. The treatment intention was mainly distributed between adjuvant (43.2%) and palliative (40.6%), with a smaller proportion of neoadjuvant (16.2%). Of the 37 patients evaluated, 19 (51.4%) received second-line therapies, and only 3 (8.1%) received third-line therapies for lung cancer. These results indicate a significant proportion of patients progressing to second-line treatments, while a smaller fraction required third-line treatments. Regarding pulmonary adverse events, only two patients (5.4%) developed grade III and IV adverse events consistent with pneumonitis.

Regarding radiotherapy, it was initiated in 19 of the 56 patients (33.9%), with the most used modality being SBRT (Stereotactic Body Radiation Therapy) in 10 cases (52.6%), followed by IMRT (Intensity-Modulated Radiation Therapy) in 9 patients (47.4%). The number of radiotherapy cycles varied from 3 to 30 depending on the patient, with a mean of 12 cycles. No radiotherapy toxicity events were documented according to the RTOG (Radiation Therapy Oncology Group) and EORTC (European Organisation for Research and Treatment of Cancer) criteria.

### 3.8. Mortality and Causes of Death

Eight of the patients in this study died during the follow-up period (14.3%). The most common causes of death were tumor progression in five patients and septic shock, stroke, and decompensated heart failure in each of the remaining three patients.

## 4. Discussion

The present study provides a comprehensive analysis of 56 patients admitted to C3 between 2 May 2022 and 22 April 2024, focusing on demographic characteristics, smoking history, comorbidities, lung cancer types, TNM classification, treatment modalities, and outcomes. Our findings are consistent with previous research and provide valuable insights into the management and prognosis of lung cancer patients [1,2,3,4,11,12].

The demographic data revealed a predominance of elderly patients with a mean age of 71.8 years. The gender distribution showed a slightly higher proportion of females (53.6%) compared to males (46.4%). These findings align with the general epidemiological trends observed in lung cancer, where age and gender are significant risk factors [11,12].

The smoking history analysis indicated that 71.4% of patients had a history of smoking, while 28.6% had never smoked. This is significantly higher than the prevalence of tobacco use in the Colombian general population, which is approximately 33% [4]. Notably, none of the patients were current smokers at the time of this study. This is significant, as smoking is a well-established risk factor for lung cancer, and smoking cessation is known to improve prognosis and reduce recurrence rates [26].

Comorbidities were prevalent among the patient cohort, with hypertension (21.4%), diabetes mellitus (14.3%), and chronic obstructive pulmonary disease (COPD) (10.7%) being the most common. The correlation between these comorbidities and the medications prescribed highlights the importance of comprehensive management in lung cancer patients. For instance, hypertension was commonly treated with antihypertensive medications such as enalapril and losartan, while diabetes mellitus management included metformin and insulin. These findings underscore the necessity of addressing comorbid conditions to optimize overall patient outcomes.

In terms of lung cancer types, adenocarcinoma was the most prevalent, accounting for 75.0% of cases, followed by squamous cell carcinoma at 19.6%. This distribution is consistent with global patterns, where adenocarcinoma is the most common histological subtype of lung cancer [15,16,17]. The TNM classification at admission and the last visit showed a progression in some patients, emphasizing the aggressive nature of the disease and the need for continuous monitoring and treatment adjustments [27].

The survival analysis revealed that 68.8% of patients survived at least one year post-diagnosis, while 31.2% succumbed within the first year. This survival rate is like or even higher than what has been reported in the literature [8,9,10]. The utilization of PET scans was high, with 94.3% of stage I–IIIA patients undergoing this diagnostic procedure, which is crucial for accurate staging and treatment planning [28,29,30]. PET scan results indicated a high rate of negative findings (72.7%), suggesting effective initial treatments or low tumor activity at the time of evaluation.

Biomolecular profiling and biomarker analysis showed most of the patients included in this study underwent profiling, with non-mutated EGFR being the most common result (69.2%). This aligns with the current understanding that EGFR mutations, although less common, have significant therapeutic implications. The presence of ALK and PDL-1 biomarkers also provides opportunities for targeted therapies, which are increasingly becoming standard care in advanced lung cancer [19,20,21,22].

Oncological management predominantly involves immunotherapy, with 91.4% of patients receiving this treatment modality. Alectinib and osimertinib were the most frequently used medications, reflecting their efficacy in targeted treatment [31,32,33,34,35,36,37,38]. Adjuvant (43.2%) and palliative (40.6%) treatments were the primary intents, highlighting the diverse therapeutic approaches based on disease stage and patient condition.

The incidence of grades III and IV pneumonitis was low (5.4%), indicating that severe pulmonary adverse events were relatively rare. This is like what has been reported in the literature [39]. Radiotherapy, particularly SBRT and IMRT, was utilized in 33.9% of patients, with a mean of 12 cycles per patient. The absence of documented radiotherapy toxicity according to RTOG and EORTC criteria suggests that these modalities were well tolerated. This is notable as it has been reported that between 10 and 30% of all patients with lung or breast cancer receiving thoracic radiotherapy develop radiation-induced pneumonitis (RIP) as a subacute treatment-associated toxicity, and they are at high risk of developing radiation-induced lung fibrosis (RILF) as late toxicity, although treatment-related death is uncommon [40]. However, it is possible that the low power of the study as well as the short follow-up period did not allow for a more accurate assessment of this outcome.

Mortality analysis revealed that 14.3% of patients died during the study period. The most common causes of death were tumor progression in five patients and septic shock, stroke, and decompensated heart failure in each of the remaining three patients. These findings emphasize the need for ongoing supportive care and early intervention to manage complications.

In the context of previous studies, our findings corroborate the established risk factors, comorbidities, and treatment outcomes in lung cancer patients. The high utilization of diagnostic and therapeutic modalities aligns with current clinical guidelines, reflecting a comprehensive approach to lung cancer management.

Despite the valuable insights provided by this study, several limitations must be acknowledged. As an observational study based on a case series and the review of medical records, it is inherently subject to selection bias, recall bias, and other limitations typical of retrospective data collection. The sample size of 56 patients, while sufficient to offer preliminary insights, limits the statistical power and may not provide a comprehensive representation of the broader population of lung cancer patients. This constraint is particularly important given the heterogeneity of lung cancer in terms of histological subtypes, stages at diagnosis, and patient demographics, which could affect the generalizability of the findings.

Additionally, the relatively short follow-up period poses a significant limitation, particularly in assessing long-term outcomes and late-onset toxicities such as radiation-induced lung fibrosis (RILF), which may take years to manifest. This limited follow-up may lead to an underestimation of the incidence of these complications, and, as a result, the conclusions about long-term efficacy and safety are necessarily incomplete. Extending the follow-up period in future studies will be essential to capture a more accurate picture of survival rates, quality of life, and the incidence of delayed adverse events.

The study’s low statistical power also restricts its ability to detect smaller effect sizes or subtle differences between subgroups, which could be critical in identifying specific patient characteristics that influence treatment outcomes. For example, variations in response to treatment based on genetic factors, comorbidities, or previous treatments might not have been fully captured due to the small sample size and limited subgroup analysis. This highlights the need for larger, multi-center studies that can enroll a more diverse cohort and provide more robust statistical analyses.

Future research should aim to address these limitations by not only incorporating larger and more heterogeneous patient populations but also adopting a prospective design. Prospective studies would mitigate the inherent biases of retrospective data collection and allow for a more controlled and systematic assessment of patient outcomes, particularly in evaluating long-term efficacy and safety. Randomized controlled trials (RCTs) would further help validate these findings, offering stronger evidence for optimizing therapeutic strategies and ensuring that the results can be applied to clinical practice with greater confidence. These efforts would ultimately contribute to refining treatment protocols and improving outcomes for lung cancer patients.

To improve outcomes for NSCLC patients, several strategies could be considered. First, enhancing early detection through expanded screening programs, especially for high-risk groups, would be crucial in reducing the presentation of late-stage NSCLC. Additionally, increasing access to comprehensive biomarker testing nationwide could enable more precise, targeted therapies, optimizing treatment efficacy. This aligns with a future plan to strengthen precision medicine in Colombia by promoting accessibility to biomolecular profiling and newer targeted therapies, potentially through collaborative networks and policy adjustments. Moreover, implementing multidisciplinary lung cancer teams and streamlining referral pathways could improve patient management and ensure timely treatment, particularly for patients in underserved regions. These approaches could collectively contribute to achieving more favorable NSCLC outcomes in Colombia.

## Figures and Tables

**Table 1:** Baseline characteristics and smoking history.

Characteristic	Value
Number of patients	56
Age range (years)	49–90
Mean age (years)	71.8
Female, n (%)	30 (53.6%)
Male, n (%)	26 (46.4%)
Ever smoked, n (%)	40 (71.4%)
Never smoked, n (%)	16 (28.6%)
Currently smoking, n (%)	0 (0%)

**Table 2:** Pathologies and medications.

Pathologies	n (%) Pathologies	Medications	n (%) Medications
Hypertension	12 (21.4%)	Atorvastatin	12 (21.4%)
Diabetes mellitus	8 (14.3%)	Omeprazole	8 (14.3%)
Chronic obstructive pulmonary disease (COPD)	6 (10.7%)	Metformin	6 (10.7%)
Ischemic heart disease	5 (8.9%)	Enalapril	5 (8.9%)
Dyslipidemia	4 (7.1%)	Levothyroxine	3 (5.4%)
Hypothyroidism	3 (5.4%)	Metoprolol	3 (5.4%)
Previous cancer	2 (3.6%)	Insulin	2 (3.6%)
Chronic renal failure	2 (3.6%)	Aspirin	2 (3.6%)
Other pathologies	14 (25.0%)	Other medications	15 (26.7%)

**Table 3:** Lung cancer types and the TNM classification.

Lung Cancer Type	n (%) Lung Cancer Type	Initial TNM Stage	n (%) Initial TNM	Current TNM Stage	n (%) Current TNM
Adenocarcinoma	42 (75.0%)	IA1	1 (1.8%)	IA1	1 (1.8%)
Squamous cell carcinoma	11 (19.6%)	IA2	10 (17.9%)	IA2	9 (16.1%)
Poorly differentiated carcinoma	2 (3.6%)	IA3	3 (5.4%)	IA3	3 (5.4%)
Infiltrating carcinoma with neuroendocrine differentiation	1 (1.8%)	IB	8 (14.3%)	IB	8 (14.3%)
		IIA	1 (1.8%)	IIA	1 (1.8%)
		IIB	6 (10.7%)	IIB	4 (7.1%)
		IIIA	6 (10.7%)	IIIA	6 (10.7%)
		IIIB	4 (7.1%)	IIIB	4 (7.1%)
		IIIC	2 (3.6%)	IIIC	2 (3.6%)
		IVA	9 (16.1%)	IVA	9 (16.1%)
		IVB	6 (10.7%)	IVB	9 (16.1%)

## Data Availability

Due to institutional policies and in compliance with the Habeas Data Law and data protection regulations applicable in Colombia, it is not possible to publicly share the corresponding database for this study. However, this information will be available upon request to other researchers.
